# Alcohol Consumption Index as a Novel Risk Marker for Hypertension

**DOI:** 10.1002/npr2.70166

**Published:** 2026-09-11

**Authors:** Ichiro Wakabayashi

**Affiliations:** ^1^ Department of Preventive Medicine School of Medicine, Hyogo Medical University Nishinomiya Japan

**Keywords:** alcohol consumption, biomarker, blood pressure, hypertension, retrospective cohort study

## Abstract

**Objectives:**

Habitual alcohol consumption is a risk factor for hypertension. The alcohol consumption index (ACI), which is defined as the product of mean corpuscular volume, gamma‐glutamyl transferase, and high‐density lipoprotein cholesterol levels in blood, has been proposed as a biomarker for estimating individual alcohol consumption. The aim of this study was to clarify the relationship between ACI and blood pressure.

**Methods:**

The participants were an occupational cohort consisting of 6 655 men (median age: 51 years). A retrospective cohort study was performed to compare variables at baseline of the study and 5 years later. The participants were categorized by frequency of alcohol intake as non‐, occasional, or regular drinkers and by amount of average daily alcohol consumption as non‐, light, or heavy drinkers. They were also classified into three tertiles according to ACI.

**Results:**

The odds ratios for incident hypertension in regular drinkers vs. nondrinkers [1.71 (95% confidence interval: 1.42 ~ 2.06)], heavy drinkers vs. nondrinkers [1.95 (1.60 ~ 2.37)], and the 3rd vs. 1st tertiles of ACI [1.81 (1.51 ~ 2.16)] were significantly higher than the reference level. The odds ratio for incident hypertension in the 3rd vs. 1st tertiles of ACI remained significant even after adjusting for blood pressure at baseline. In groups of individuals who changed after the 5‐year follow‐up from regular or heavy drinkers to nondrinkers and from the 3rd tertile of ACI to the 1st tertile, systolic and diastolic blood pressure levels were significantly lower after the follow‐up than at baseline.

**Conclusion:**

ACI, as well as alcohol intake, was associated with the risk of hypertension. Decreases in ACI and alcohol consumption after the 5‐year follow‐up were associated with reductions in blood pressure. These findings suggest that ACI is a useful biomarker of alcohol‐related hypertension.

**Tweetable Abstract:**

The alcohol consumption index (ACI), defined as the product of GGT, HDL, and MCV, is associated with blood pressure and incident hypertension and may be a useful biomarker for prevention of alcohol‐induced hypertension.

## Abstract 2

The alcohol consumption index (ACI), defined as the product of GGT, HDL, and MCV, is a new marker of alcohol consumption. Similar to self‐reported alcohol consumption, ACI was associated with incident hypertension. Decreases in ACI and self‐reported alcohol consumption after 5‐year follow‐up were associated with reductions in blood pressure. Therefore, ACI may be a useful biomarker of alcohol‐related hypertension.

## Graphical abstract

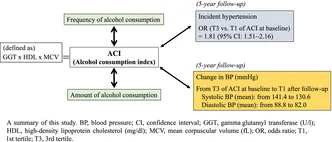

## 1 Introduction

Habitual alcohol consumption is a risk factor for chronic hypertension and subsequent stroke [1]. Previous meta‐analyses demonstrated that the risk of hypertension was higher in men and women who consumed excessive amounts of alcohol than in those who did not consume, and a dose–response relationship between alcohol consumption and the risk of hypertension was observed in men [2, 3, 4, 5, 6, 7]. The recommended daily alcohol intake for preventing hypertension is generally less than 20 g for men and 10 g for women [8].

In epidemiological studies, underreporting of individual alcohol consumption is a recognized problem and may result from inaccurate reporting by respondents and the omission of heavier drinkers from the study samples. The average coverage of alcohol consumption was reported to be 61.71%, although it greatly differed among countries [9]. The average coverage of alcohol consumption in 23 European countries was reportedly 36.5% [10], and that in South Africa was less than 20% [11]. Therefore, blood markers that reflect average daily alcohol intake are useful for estimating alcohol consumption and may be applied in health promotion strategies for preventing alcohol‐related diseases, including hypertension. Conventional blood markers of alcohol consumption include mean corpuscular volume (MCV), gamma‐glutamyl transferase (GGT), and high‐density lipoprotein (HDL) cholesterol, which have been reported in healthy cohorts [12, 13, 14] and in patients with alcohol use disorder (AUD) [15, 16, 17, 18]. The alcohol consumption index (ACI), defined as the product of these three variables, has recently been proposed as a marker of alcohol consumption and has been shown to be more strongly associated with the frequency and amount of alcohol consumption reported in self‐administered questionnaires than each variable alone [19]. However, it remains unknown whether and how ACI is related to blood pressure and whether it is useful for the prevention of hypertension.

The purpose of this study was therefore to investigate the relationships between ACI and blood pressure and the risk of hypertension. In addition to the cross‐sectional analysis of baseline data, incident hypertension and changes in blood pressure were analyzed in relation to alcohol consumption and ACI using a retrospective analysis.

## 2 Methods

### 2.1 Study Population

The initial participants comprised 6 764 Japanese men who underwent workplace health checkups in Yamagata Prefecture, Japan. Five years after the baseline survey, each participant underwent subsequent health assessments at their workplaces. The participants were working at various kinds of companies including construction, manufacturing, information and communications, transport, wholesale and retail trade, eating and drinking places, accommodations, and services. This study was approved by the Hyogo College of Medicine Ethics Committee (No. 3003 in 2020). Participants with a history of current therapy for anemia (*n* = 26) and/or with a history of current therapy for liver disease (*n* = 85) at baseline and/or five years after the baseline survey were excluded from the study. Consequently, 6 655 participants were included in the analysis.

### 2.2 Information on Alcohol Consumption

Histories of cigarette smoking, alcohol consumption, regular exercise, illness, and therapy for illness were surveyed using a self‐administered paper questionnaire. Average alcohol consumption per week was recorded in the questionnaire. The frequency of habitual alcohol consumption was assessed with the question, “How frequently do you drink alcohol?” The frequency of weekly alcohol consumption was categorized as “every day” (regular drinkers), “sometimes” (occasional drinkers), and “never” (nondrinkers). The next question was “What amount of alcohol per day do you consume?” In the questionnaire, drinkers were classified using “go”, a traditional Japanese unit of sake (rice wine). One “go” corresponds to approximately 22 g of ethanol. The amounts of other alcoholic beverages, including beer, wine, whisky, and shochu (a traditional Japanese distilled spirit), were converted and expressed as units of go. One go corresponds to approximately 180 mL of sake, 500 mL of beer, 240 mL of wine, 60 mL of whisky, and 80 mL of shochu. The response categories for the above question on alcohol intake were “null”, “less than one go (about 22 grams of alcohol)”, “one go or more and less than two go”, “two go or more and less than three go”, and “three go or more”. Heavy drinkers were defined as those with daily alcohol consumption of ≥ 22 g of alcohol (one “go”) according to the recommendation by the Japanese Society of Hypertension (JSH2025) that daily alcohol consumption should be restricted to ≤ 20 ~ 30 mL in men from the viewpoint of prevention of hypertension [20]. Light drinkers were defined as those consuming less than one go (< 22 g of alcohol) per day. When the amount of alcohol consumption was analyzed, occasional drinkers were excluded from the analysis because their accurate average daily alcohol consumption is difficult to determine.

### 2.3 Information on Other Lifestyles

Regarding smoking status, participants were first asked in the questionnaire, “Are you a habitual cigarette smoker?” Cigarette smokers were defined as participants who had smoked for six months or longer and had smoked during the past month or longer. Participants who were smokers were then further asked, “What is your average cigarette consumption per day?” The response categories for this question were “20 or fewer cigarettes per day”, “21 or more and 40 or fewer cigarettes per day”, and “41 or more cigarettes per day”. Because the percentage of subjects with very heavy cigarette consumption (41 or more cigarettes per day) was very low [0.62% (*n* = 41)], they were included in the heavy smoker group. The subjects were then divided into three groups: Nonsmokers, light smokers (20 or fewer cigarettes per day), and heavy smokers (21 or more cigarettes per day). Subjects with a habit of regular exercise were defined as those who exercised almost every day for 30 min or more per day.

### 2.4 Measurements of Variables Used in This Study

Height and body weight were measured while participants wore light clothing during the health examination, and body mass index (BMI) was calculated as weight in kilograms divided by the square of height in meters. Blood pressure was recorded at rest in a seated position using an oscillometric device (TM‐2580; A&D Co. Ltd., Tokyo, Japan). Hypertension was defined as systolic blood pressure ≥ 140 mmHg and/or diastolic blood pressure ≥ 90 mmHg [21]. Individuals receiving medication for hypertension were also regarded as having hypertension.

Fasting blood samples were collected from each subject in the morning. Erythrocyte count and hematocrit in the blood were measured using the direct current sheath flow detection method and the erythrocyte high pulse‐wave detection method, respectively. Concentrations of HDL cholesterol, LDL cholesterol, triglycerides, and GGT in the serum were measured using enzymatic methods with the commercial kits Cholestest N‐HDL, Cholestest LDL, Pureauto S TG‐N, and Pureauto S γ‐GT (Sekisui Medical Co. Ltd., Tokyo, Japan), respectively. The ACI was calculated as the product of MCV, GGT, and HDL cholesterol [19]. Hemoglobin A_1c_ was measured by the NGSP (National Glycohemoglobin Standardization Program)‐approved technique using the latex cohesion method with a commercial kit (Determiner HbA_1c_, Kyowa Medex, Tokyo, Japan). High LDL cholesterol, high triglycerides, and diabetes were defined as LDL cholesterol of ≥ 140 mg/dL, triglycerides of ≥ 150 mg/dL, and hemoglobin A_1c_ of ≥ 6.5%. Individuals receiving drug therapy for diabetes were also included in the above definition of diabetes.

### 2.5 Statistical Analysis

Continuous variables were summarized as means with 95% confidence intervals or standard errors and medians with interquartile ranges, as appropriate. Categorical variables were summarized as frequencies and percentages. Participants were sorted by ACI levels in ascending order, and then divided into three nearly equal‐sized tertiles. The cut‐off values for the ACI tertiles between the 1st and 2nd tertiles and between the 2nd and 3rd tertiles were 150 300 and 285 700, respectively, at baseline of the study and were 147 250 and 282 300, respectively, after following up for 5 years. Continuous variables were compared between paired observations at baseline and 5 years later using the paired *t*‐test. Continuous variables at baseline were also compared among the three groups of alcohol consumption or the tertiles of ACI using analysis of variance (ANOVA) followed by Scheffé's F‐test as a post hoc test in univariable analysis and using analysis of covariance (ANCOVA) followed by Student's *t*‐test with Bonferroni's multiplicity correction in multivariable analysis. GGT and ACI levels, which did not exhibit a normal distribution, were compared after normalization by base‐10 logarithmic transformation. Categorical variables were compared using Pearson's chi‐square test. The impact of baseline alcohol consumption and ACI on incident hypertension was analyzed using a logistic regression model, and crude and adjusted odds ratios with their corresponding 95% confidence intervals were estimated in the participants after excluding those with hypertension at baseline of the study (*n* = 3 063). In the multivariable analyses (ANCOVA and multivariable logistic regression), adjustments were made for age, BMI, and histories of smoking and regular exercise. Additionally, a history of medication therapy for hypertension and metabolic factors, such as diabetes, dyslipidemia, and a history of lipid‐lowering therapy, were adjusted for as appropriate in ANCOVA. Discrimination for predicting incident hypertension was assessed using C‐statistics and DeLong's test. All probability (*p*) values were two‐sided, with statistical significance defined as *p* < 0.05. Statistical analyses were performed using IBM SPSS Statistics for Windows, Version 25.0 (IBM Corp., Armonk, NY, USA).

## 3 Results

### 3.1 Comparison of Each Variable of the Participants at Baseline of the Study and 5 Years Later

Table 1 shows the characteristics of the participants and a comparison of each variable at baseline and 5 years later. The proportions of smokers and individuals with a habit of regular exercise were significantly lower and higher, respectively, 5 years later than at baseline. Systolic blood pressure, diastolic blood pressure, and the proportions of individuals with hypertension, individuals with a history of medication therapy for hypertension, and individuals with a history of blood lipid‐lowering therapy were significantly higher 5 years later than at baseline. Hematocrit and MCV were significantly higher, and erythrocyte count was significantly lower 5 years later than at baseline. HDL cholesterol, GGT, and ACI were slightly but significantly lower 5 years later than at baseline.

**TABLE 1:** Comparison of each variable of the participants at baseline of study and 5 years later.

Variable	Baseline	5 years later
Number	6 655	6 655
Age (years)	51 (44, 57)	56 (49, 62)**
Frequency of alcohol intake		
Nondrinkers	1 468 (22.1%)	1 576 (23.7%)
Occasional drinkers	2 152 (32.3%)	2 095 (31.5%)
Regular drinkers	3 035 (45.6%)	2 984 (44.8%)
Amount of alcohol intake^a^		
Nondrinkers	1 468 (32.6%)	1 576 (34.6%)
Light drinkers	655 (14.5%)	562 (12.3%)
Heavy drinkers	2 380 (52.9%)	2 422 (53.1%)
Smoking		
Nonsmokers	3 620 (54.4%)	4 037 (60.7%)**
Light smokers	2 476 (37.2%)	2 216 (33.3%)**
Heavy smokers	559 (8.4%)	402 (6.0%)**
Regular exercise	16.9	1262 (19.0%)**
Height (cm)	170.8 (170.6 ~ 170.9)	170.2 (170.1 ~ 170.4)**
Body weight (kg)	70.3 (70.0 ~ 70.6)	70.6 (70.4 ~ 70.9)**
BMI (kg/m^2^)	24.10 (24.01 ~ 24.19)	24.36 (24.27 ~ 24.45)**
SBP (mmHg)	131.5 (131.1 ~ 131.9)	132.4 (131.9 ~ 132.8)**
DBP (mmHg)	82.5 (82.2 ~ 82.8)	84.0 (83.8 ~ 84.3)**
Hypertension	46.0	58.3**
Therapy for hypertension	21.3	33.9**
Lipid‐lowering therapy	8.1	14.4**
Erythrocyte count (x 10^4^/μL)	494.2 (493.2 ~ 495.1)	486.2 (485.1 ~ 487.2)**
Hematocrit (%)	45.21 (45.14 ~ 45.28)	45.97 (45.88 ~ 46.04)**
MCV (fL)	91.71 (91.60 ~ 91.81)	94.79 (94.68 ~ 94.90)**
GGT (U/L)^b^	39 (25, 65)	37 (24, 63)**
HDL cholesterol (mg/dl)	59.87 (59.51 ~ 60.24)	59.03 (58.67 ~ 59.39)**
ACI^b^	201 199	198 819
	(128 226, 354 205)	(127 723, 345 797)**

*Note:* Numbers, frequency, proportions, means with 95% confidence intervals, and medians with interquartile ranges of the variables are shown. Asterisks denote significant differences between the values at baseline and 5 years later (*, *p* < 0.05; **, *p* < 0.01).

^a^ Occasional drinkers were excluded from the analysis of alcohol intake amount.

^b^ GGT and ACI levels were compared after logarithmic transformation.

### 3.2 Comparison of Log‐Transformed ACI at Baseline Among Different Drinker Groups Divided by Frequency and Amount of Alcohol Consumption

**FIGURE 1:**
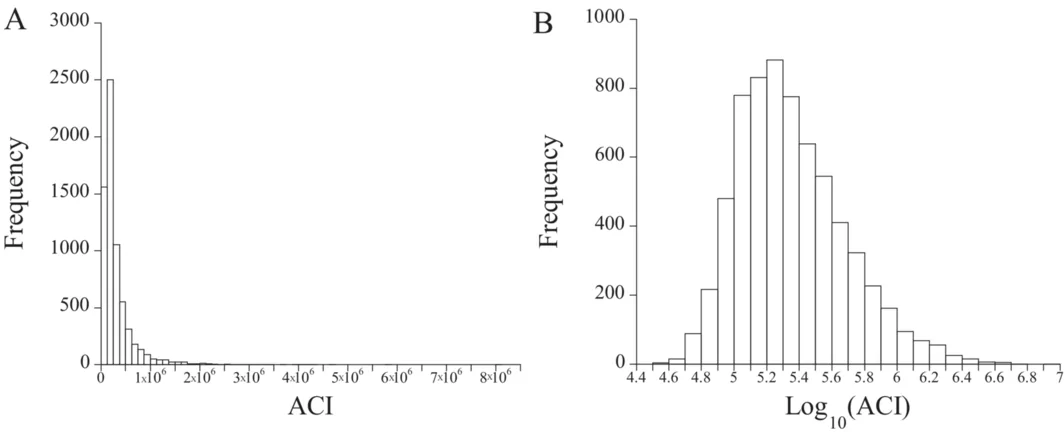
Histograms of ACI (A) and base‐10 log‐transformed ACI (B).

**FIGURE 2:**
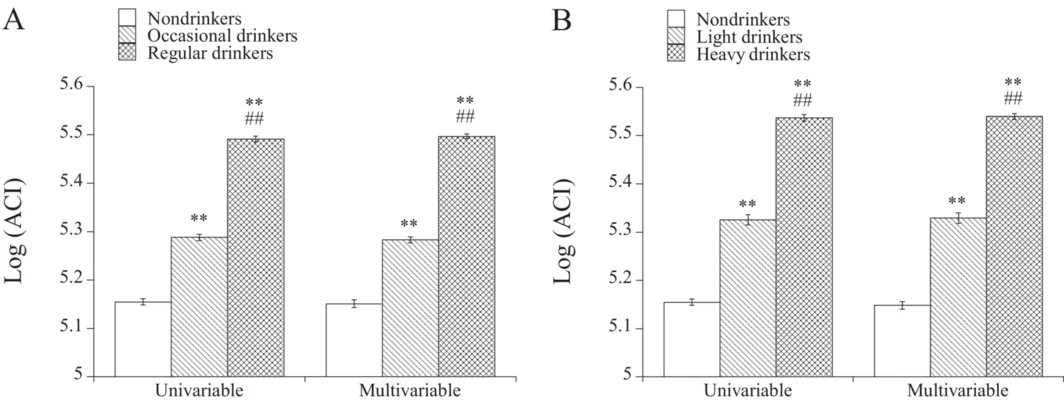
Comparison of log‐transformed ACI in different groups divided by frequency of alcohol intake (A) and amount of alcohol consumption (B). Means with standard errors of log‐transformed ACI are shown. Age, BMI, current therapy for hypertension, and habits of smoking and regular exercise were adjusted for in the multivariable analysis. Symbols denote significant differences from nondrinkers (**, *p* < 0.01) and occasional or light drinkers (##, *p* < 0.01).

As shown in Figure 1, ACI did not show a normal distribution and was therefore used after normalization by base‐10 logarithmic transformation. In both the univariable and multivariable analyses with adjustment for age, BMI, current therapy for hypertension, and habits of smoking and regular exercise, log‐transformed ACI tended to be higher with increasing frequency of alcohol intake (Figure 2A) and increasing amount of alcohol consumption (Figure 2B).

### 3.3 Comparison of Blood Pressure Levels at Baseline Among Groups Divided by Frequency and Amount of Alcohol Consumption and Tertiles of ACI

Systolic and diastolic blood pressure levels were significantly higher in regular drinkers than in nondrinkers. Systolic blood pressure was not significantly different between occasional drinkers and nondrinkers, whereas diastolic blood pressure was slightly but significantly higher in occasional drinkers than in nondrinkers (Table 2). Systolic and diastolic blood pressure levels were significantly higher in heavy drinkers than in nondrinkers. Systolic blood pressure was not significantly different between light drinkers and nondrinkers, whereas diastolic blood pressure was slightly but significantly higher in light drinkers than in nondrinkers (Table 2). Systolic and diastolic blood pressure levels were significantly higher in the 2nd and 3rd tertile groups of ACI than in the 1st tertile group and were significantly higher in the 3rd tertile group of ACI than in the 2nd tertile group (Table 2).

**TABLE 2:** Comparison of blood pressure in different groups classified by frequency of alcohol consumption, amount of alcohol consumption, and tertiles of ACI.

	Systolic blood pressure (mmHg) Univariable	Multivariable	Diastolic blood pressure (mmHg) Univariable	Multivariable
Frequency of drinking				
Non	128.9 (128.0 ~ 129.8)	128.9 (128.0 ~ 129.8)	80.2 (79.6 ~ 80.9)	80.1 (79.5 ~ 80.7)
Occasional	129.6 (128.9 ~ 130.3)	129.5 (128.8 ~ 130.3)	81.6 (81.1 ~ 82.1)**	81.3 (80.8 ~ 81.8)**
Regular	134.1 (133.4 ~ 134.7)**, ##	134.1 (133.5 ~ 134.7)**, ##	84.3 (83.9 ~ 84.7)**, ##	84.6 (84.2 ~ 85.0)**, ##
Amount of drinking				
Non	128.9 (128.0 ~ 129.8)	128.9 (128.0 ~ 129.8)	80.2 (79.6 ~ 80.9)	79.9 (79.3 ~ 80.5)
Light	130.6 (129.3 ~ 132.0)	131.1 (129.8 ~ 132.4)*	82.1 (81.2 ~ 83.0)**	82.4 (81.5 ~ 83.3)**
Heavy	135.0 (134.3 ~ 135.7)**, ##	134.9 (134.2 ~ 135.6)**, ##	84.9 (84.4 ~ 85.4)**, ##	85.1 (84.6 ~ 85.5)**, ##
Tertiles of ACI				
1st	126.9 (126.2 ~ 127.6)	128.1 (127.4 ~ 128.9)	78.9 (78.4 ~ 79.4)	79.5 (79.0 ~ 79.9)
2nd	131.6 (130.9 ~ 132.4)**	131.4 (130.7 ~ 132.1)**	82.8 (82.3 ~ 83.3)**	82.5 (82.1 ~ 83.0)**
3rd	136.0 (135.2 ~ 136.7)**, ##	135.4 (134.7 ~ 136.1)**, ##	85.9 (85.4 ~ 86.4)**, ##	85.6 (85.1 ~ 86.1)**, ##

*Note:* Means with 95% confidence intervals of systolic and diastolic blood pressure are shown. Age, BMI, medication therapy for hypertension, habit of smoking, and habit of regular exercise were adjusted for in the multivariable analysis. Symbols denote significant differences from nondrinkers or the 1st tertile of ACI (*, *p* < 0.05; **, *p* < 0.01) and occasional drinkers, light drinkers or the 2nd tertile of ACI (##, *p* < 0.01).

The relationships between ACI and blood pressure were also investigated by multivariable analysis with adjustment for metabolic factors such as diabetes mellitus, dyslipidemia (high triglycerides and/or high LDL cholesterol), and lipid‐lowering therapy. In multivariable analysis with the above adjustment, mean levels with 95% confidence intervals of systolic blood pressure (mmHg) in the 1st, 2nd and 3rd tertile groups of ACI were 127.3 (126.6 ~ 128.0), 131.5 (130.8 ~ 132.3) and 135.6 (134.9 ~ 136.4), respectively (*p* < 0.01), and mean levels of diastolic blood pressure (mmHg) in the 1st, 2nd and 3rd tertile groups of ACI were 79.2 (78.7 ~ 79.7), 82.7 (82.2 ~ 83.2) and 85.7 (85.2 ~ 86.2), respectively (*p* < 0.01). Thus, the positive associations of ACI with systolic and diastolic blood pressure levels were not influenced by the metabolic factors such as dyslipidemia and diabetes.

### 3.4 Comparison of Blood Pressure Levels at Baseline and 5 Years Later in Each Pair of Groups Divided by Frequency of Alcohol Intake

**FIGURE 3:**
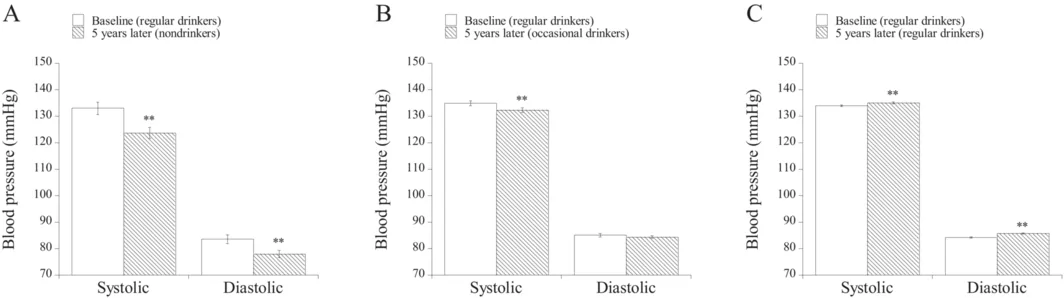
Comparison of systolic and diastolic blood pressure levels at baseline and 5 years later in each pair of groups divided by frequency of alcohol intake. (A) Regular drinkers at baseline and nondrinkers 5 years later (*n* = 76); (B) Regular drinkers at baseline and occasional drinkers 5 years later (*n* = 373); (C) Regular drinkers at baseline and regular drinkers 5 years later (*n* = 2 586). Means with standard errors of blood pressure are shown. Asterisks denote significant differences from the levels at baseline (**, *p* < 0.01).

Figure 3 shows a comparison of blood pressure at baseline and 5 years later in regular drinkers at baseline. In the group of nondrinkers 5 years later, systolic and diastolic blood pressure levels were significantly lower 5 years later than at baseline (Figure 3A). In the group of occasional drinkers 5 years later, systolic blood pressure was slightly but significantly lower 5 years later than at baseline, whereas diastolic blood pressure was not significantly different at baseline and 5 years later (Figure 3B). In the group of regular drinkers 5 years later, systolic and diastolic blood pressure levels were slightly but significantly higher 5 years later than at baseline (Figure 3C).

### 3.5 Comparison of Blood Pressure Levels at Baseline of the Study and 5 Years Later in Each Pair of Groups Divided by Amount of Alcohol Consumption

**FIGURE 4:**
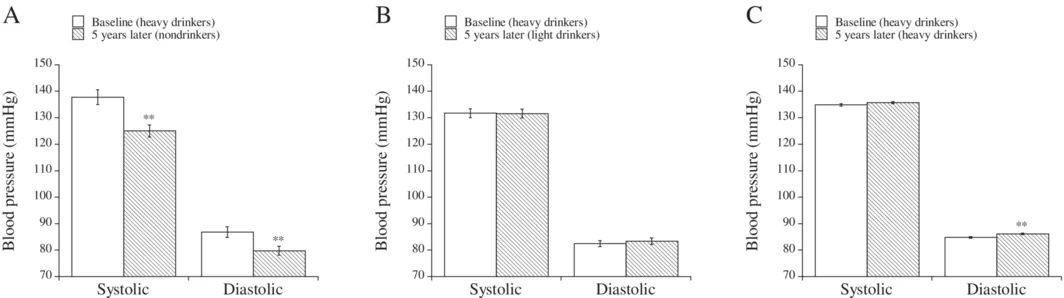
Comparison of systolic and diastolic blood pressure levels at baseline of this study and 5 years later in each pair of groups divided by amount of alcohol consumption. (A) Heavy drinkers at baseline and nondrinkers 5 years later (*n* = 50); (B) Heavy drinkers at baseline and light drinkers 5 years later (*n* = 118); (C) Heavy drinkers at baseline and heavy drinkers 5 years later (*n* = 1 981). Means with standard errors of blood pressure are shown. Asterisks denote significant differences from the levels at baseline (**; *p* < 0.01).

Figure 4 shows a comparison of blood pressure at baseline and 5 years later in heavy drinkers at baseline. In the group of nondrinkers 5 years later, systolic and diastolic blood pressure levels were significantly lower 5 years later than at baseline (Figure 4A). In the group of light drinkers 5 years later, systolic and diastolic blood pressure levels were not significantly different at baseline and 5 years later (Figure 4B). In the group of heavy drinkers 5 years later, diastolic blood pressure was slightly but significantly higher 5 years later than at baseline, whereas systolic blood pressure was not significantly different at baseline and 5 years later (Figure 4C).

### 3.6 Comparison of Blood Pressure at Baseline of the Study and 5 Years Later in Each Pair of Groups Divided by Tertiles of ACI

**FIGURE 5:**
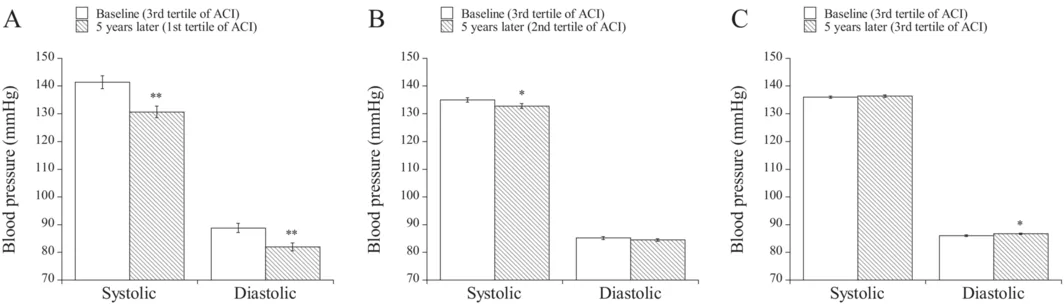
Comparison of systolic and diastolic blood pressure levels at baseline of this study and 5 years later in each pair of the groups divided by tertiles of ACI. (A) 3rd tertile of ACI at baseline and 1st tertile of ACI 5 years later (*n* = 65); (B) 3rd tertile of ACI at baseline and 2nd tertile of ACI 5 years later (*n* = 484); (C) 3rd tertile of ACI at baseline and 3rd tertile of ACI 5 years later (*n* = 1 670). Means with standard errors of blood pressure are shown. Asterisks denote significant differences from the levels at baseline (*, *p* < 0.05; **, *p* < 0.01).

Figure 5 shows a comparison of blood pressure at baseline and 5 years later in the 3rd tertile group of ACI at baseline. In the group of the 1st tertile of ACI 5 years later, systolic and diastolic blood pressure levels were significantly lower 5 years later than at baseline (Figure 5A). In the group of the 2nd tertile of ACI 5 years later, systolic blood pressure was slightly but significantly lower than at baseline, whereas diastolic blood pressure was not significantly different at baseline and 5 years later (Figure 5B). In the group of the 3rd tertile of ACI 5 years later, diastolic blood pressure was slightly but significantly higher than at baseline, whereas systolic blood pressure was not significantly different at baseline and 5 years later (Figure 5C).

### 3.7 Comparison of ACI in the Normotensive and Hypertensive Groups

In both the normotensive and hypertensive groups at baseline of the study, log‐transformed ACI levels at baseline and 5 years later were significantly higher in the hypertensive group than in the normotensive group after a 5‐year follow‐up (Table 3). Interestingly, in the group with hypertension at baseline and normal blood pressure after the follow‐up, log‐transformed ACI levels tended to be lower after the follow‐up than at baseline, even though age‐dependent elevation of blood pressure would be expected during 5‐year follow‐up. This decrease in ACI may reflect reduction of alcohol consumption.

**TABLE 3:** Comparison of ACI at baseline of the study and after 5‐year follow‐up in the groups with and without hypertension.

Blood pressure	Blood pressure	Log(ACI)	Log(ACI)
At baseline	5 years later	At baseline	5 years later
Normotensive	Normotensive	5.267 (5.255 ~ 5.279)	5.270 (5.258 ~ 5.282)
Normotensive	Hypertensive	5.338 (5.320 ~ 5.356)**	5.393 (5.374 ~ 5.412)**
Hypertensive	Normotensive	5.371 (5.331 ~ 5.411)	5.235 (5.158 ~ 5.311)
Hypertensive	Hypertensive	5.430 (5.417 ~ 5.442)**	5.407 (5.395 ~ 5.420)**

*Note:* Means with 95% confidence intervals of base‐10 log‐transformed ACI are shown. Age, BMI, current therapy for hypertension, and habits of smoking and regular exercise were adjusted. Asterisks denote significant differences from the corresponding normotensive groups after 5‐year follow‐up (**, *p* < 0.01).

### 3.8 Relationships Between Alcohol Consumption and Incident Hypertension

Participants who had been diagnosed with hypertension at baseline [*n* = 3 063 (46.0%)] were excluded from the logistic regression analysis. Following the 5‐year follow‐up period, 1 109 participants (30.9%) were newly diagnosed with hypertension. In both the univariable and multivariable analyses, the odds ratios for incident hypertension of regular drinkers vs. nondrinkers and heavy drinkers vs. nondrinkers were significantly higher than the reference level of 1.00. The odds ratio for hypertension in occasional drinkers vs. nondrinkers was also significantly higher than the reference level in the multivariable analysis. The odds ratio tended to increase with increasing frequency and amount of alcohol consumption (Table 4).

**TABLE 4:** Odds ratios for incident hypertension of each group according to frequency or amount of alcohol consumption vs. nondrinkers and each tertile group of ACI vs. the 1st tertile.

	OR for hypertension (Univariable)	OR for hypertension (Multivariable)
Frequency of alcohol intake		
Non	1.00	1.00
Occasional	1.21 (0.99 ~ 1.46)	1.22 (1.00 ~ 1.49)*
Regular	1.71 (1.42 ~ 2.06)**	1.83 (1.51 ~ 2.22)**
Amount of alcohol intake^a^		
Non	1.00	1.00
Light	1.16 (0.89 ~ 1.52)	1.27 (0.96 ~ 1.68)
Heavy	1.95 (1.60 ~ 2.37)**	2.09 (1.71 ~ 2.56)**
ACI		
1st tertile	1.00	1.00
2nd tertile	1.40 (1.18 ~ 1.65)**	1.24 (1.05 ~ 1.48)*
3rd tertile	1.81 (1.51 ~ 2.16)**	1.68 (1.40 ~ 2.02)**

*Note:* Odds ratios (ORs) with their 95% confidence intervals are shown. Asterisks denote significant differences from the reference level of 1.00 (*, *p* < 0.05; **, *p* < 0.01).

^a^ Occasional drinkers were excluded from the analysis of alcohol intake amount. Age, BMI, habit of smoking, and habit of regular exercise were adjusted for in the multivariable analysis.

### 3.9 Relationship Between ACI and Incident Hypertension

Table 4 also shows the odds ratios for hypertension according to tertile of ACI. In both the univariable and multivariable analyses, the odds ratios of the 2nd and 3rd tertiles of ACI vs. the 1st tertile were significantly higher than the reference level, and the odds ratio tended to increase with increasing tertile.

## 4 Discussion

ACI was positively associated with the frequency and amount of alcohol consumption, which was independent of age, BMI, and habits of smoking and regular exercise. ACI, as well as the frequency and amount of alcohol consumption, was positively associated with blood pressure independent of a history of medication therapy for hypertension, in addition to the above possible confounding factors and metabolic factors such as diabetes and dyslipidemia. Thus, ACI has been suggested to be a marker of alcohol consumption in relation to blood pressure. To further investigate the relationship between ACI and blood pressure, a retrospective cohort analysis was performed by following up the participants for five years. In the logistic regression analysis, the odds ratios for incident hypertension vs. the 1st tertile of ACI tended to increase with increasing tertiles and were comparable to the corresponding odds ratios in drinkers according to the frequency and amount of alcohol consumption, using nondrinkers as the reference group. The odds ratio for hypertension in the 3rd vs. 1st tertiles of ACI remained significant even after adjusting for systolic blood pressure at baseline [odds ratio with 95% confidence interval: 1.40 (1.15–1.69), *p* < 0.01], suggesting elevation of blood pressure during the 5‐year follow‐up in relation to ACI reflecting alcohol consumption. As expected, in the group of individuals who abstained from alcohol during the follow‐up, systolic and diastolic blood pressure levels were significantly lower after follow‐up than at baseline (Figures 3A and 4A). These results are plausible because a reduction in alcohol intake was demonstrated to lower blood pressure in a meta‐analysis [22]. Similarly, in individuals belonging to the 3rd tertile of ACI at baseline and the 1st tertile five years later, the systolic and diastolic blood pressure levels were significantly lower after follow‐up than at baseline (Figure 5A). Thus, a decrease in ACI after the 5‐year follow‐up was associated with lower blood pressure, which coincides with the association between changes in alcohol consumption and blood pressure after follow‐up (Figures 3A and 4A) and therefore supports the concept that ACI is a reasonable marker of alcohol consumption.

Since blood pressure increases with age, the blood pressure levels were, as expected, significantly higher after the 5‐year follow‐up (median age, 56 years) than those at the baseline (median age, 51 years) (Table 1). This may explain the slight but significant increases in diastolic blood pressure after follow‐up in the regular drinker group both at baseline and after follow‐up (Figure 3C), in the heavy drinker group both at baseline and after follow‐up (Figure 4C), and in the 3rd tertile of ACI both at baseline and after follow‐up (Figure 5C). Even with the expected age‐dependent elevation of blood pressure, significant decreases in systolic and diastolic blood pressure levels were found in the groups of regular and heavy drinkers at baseline and nondrinkers after follow‐up (Figures 3A and 4A) and in the group of the 3rd tertile of ACI at baseline and the 1st tertile of ACI after follow‐up (Figure 5A), suggesting the effectiveness of reducing alcohol consumption for the prevention of hypertension. In a recent meta‐analysis with a median follow‐up of 5.3 years, the average increases in systolic and diastolic blood pressure levels were 4.90 and 3.10 mmHg, respectively, for 48 g of daily alcohol consumption compared with no alcohol consumption [23]. In another recent meta‐analysis examining the relationship between alcohol intake and hypertension, the risk ratios for 0, 24, 36 and 48 g of alcohol per day were 0.89, 1.11, 1.22 and 1.33, respectively, with 12 g per day as the reference value [7]. Therefore, the results of the present study appear reasonable compared with the findings of the above previous studies.

ACI has several clinical implications. In the clinical setting of ACI, regular drinkers with hypertension at baseline who reported quitting alcohol consumption after a 5‐year follow‐up have a possibility of false reports. In such cases, a change in the ACI can be used to confirm quitting alcohol consumption. In the present study, 41 individuals were hypertensive regular drinkers at baseline and reported quitting alcohol consumption at the 5‐year follow‐up. Among them, eight individuals (19.5%) showed no change in the tertile group of ACI (from 2nd tertile to 2nd tertile or from 3rd tertile to 3rd tertile), while 27 individuals showed a decrease in the tertile number of ACI. Six individuals belonged to the 1st tertile of ACI both at baseline and five years later. Thus, among hypertensive regular drinkers at baseline and nondrinkers after the 5‐year follow‐up, approximately 20% were suspected to have made a false report on alcohol consumption in the present study. As another utility of ACI, there is a possibility of underreported alcohol consumption at screening for health check‐ups if individuals who self‐reported as nondrinkers or light drinkers show high ACI. Then, blood pressure levels were compared among the tertile groups of ACI in those who were self‐reported as nondrinkers (*n* = 1 468) at baseline. The numbers in the 1st, 2nd, and 3rd tertile groups were 871, 433, and 164, respectively. Thus, 11.2% of non‐drinkers were classified into the highest tertile group of ACI. Mean systolic and diastolic blood pressure levels were significantly higher in the 2nd and 3rd tertile groups than in the 1st tertile group and tended to be higher with an increase in tertile (systolic blood pressure [mmHg]: 127.5 (126.3 ~ 128.7) [1st tertile], vs. 130.2 (128.6 ~ 131.8) [2nd tertile] vs. 133.4 (130.4 ~ 136.4) [3rd tertile]; diastolic blood pressure [mmHg]: 78.8 (78.0 ~ 79.6) [1st tertile], vs. 81.9 (80.8 ~ 83.1) [2nd tertile] vs. 83.5 (81.5 ~ 85.5) [3rd tertile]). Therefore, self‐reported non‐drinkers with high ACI levels (especially hypertensive nondrinkers) are suspected of underreporting their alcohol consumption. Thus, there is a possibility that individuals who underreport self‐reported alcohol intake can be detected using ACI; however, this hypothesis needs to be clarified in further studies using larger cohorts.

Furthermore, discrimination by ACI and self‐reported alcohol consumption for predicting incident hypertension was assessed using C‐statistics. The C‐statistics with 95% confidence intervals for ACI (three tertile groups) and frequency of alcohol consumption (three groups of nondrinkers, occasional drinkers, and regular drinkers) were 0.565 (0.544 ~ 0.585) and 0.559 (0.539 ~ 0.579), respectively, and the C‐statistics for ACI and amount of alcohol consumption (three groups of nondrinkers, light drinkers, and heavy drinkers) were 0.569 (0.544 ~ 0.593) and 0.581 (0.556 ~ 0.606), respectively. In the latter analysis, occasional drinkers were excluded because their correct amounts of alcohol consumption were difficult to determine. Thus, both ACI and self‐reported alcohol consumption showed similar low accuracy for predicting incident hypertension. Moreover, the variables consisting of both ACI and self‐reported alcohol consumption were prepared by further classification of each drinker group into three tertile groups of ACI (total of nine groups). The C‐statistic for the variable consisting of ACI and frequency of alcohol consumption and that for the variable consisting of ACI and amount of alcohol consumption were 0.576 (0.556 ~ 0.597) and 0.593 (0.569 ~ 0.618), respectively, which were not significantly different from the corresponding C‐statistics for ACI alone. Thus, the combination of ACI and self‐reported alcohol consumption did not provide additional value beyond ACI alone for the prediction of hypertension. This may be explained by the fact that ACI strongly reflects alcohol consumption and their associations with the risk of hypertension largely overlap.

There is a possibility of confounding by factors changing during the 5‐year follow‐up for the relationships of alcohol consumption and ACI with incident hypertension. The percentages of the three groups of changes in each variable of antihypertensive medication, BMI, smoking or exercise during the 5‐year follow‐up were compared in the groups of changes in ACI and amount of alcohol consumption. In the three groups in the 3rd tertile of ACI at baseline, among the above variables, only the change in BMI, but not changes in antihypertensive medication, smoking or exercise, showed a significant relationship with the changes in ACI tertile after the 5‐year follow‐up, while in the heavy drinker group at baseline, changes in any of the above four variables did not show significant relationships with the change in amount of alcohol consumption after the 5‐year follow‐up (data not shown). Then, multiple regression analysis for the relationship between ACI and incident hypertension was performed with adjustment for changes in BMI over 5 years. The odds ratios with 95% confidence intervals for hypertension of the 2nd and 3rd vs. 1st tertiles of ACI with adjustment for changes in BMI during 5‐year follow‐up were 1.42 (1.20 ~ 1.68) and 1.87 (1.56 ~ 2.23), respectively, which were comparable to the corresponding crude odds ratios shown in Table 4. Therefore, it was concluded that changes in antihypertensive medication, BMI, smoking, or exercise during the 5‐year follow‐up period may not have affected the association between ACI and incident hypertension.

It is also possible that lipid‐lowering therapy affects the relationship between ACI and hypertension. The proportion of participants receiving lipid‐lowering therapy increased from 8.1% to 14.4% during the 5‐year follow‐up. When comparing ACI and HDL‐cholesterol at baseline of the study between two groups with and without receiving lipid‐lowering therapy with adjustment for age, BMI, and histories of smoking, alcohol consumption, and regular exercise, log‐transformed ACI and HDL cholesterol were slightly but significantly higher and lower, respectively, in the group with the therapy than in that without the therapy (log‐transformed ACI: 5.386 [5.353 ~ 5.419] for those with the therapy vs. 5.349 [5.342 ~ 5.356] for those without the therapy; HDL cholesterol (mg/dl): 57.7 [56.2 ~ 59.2] for those with the therapy vs. 59.9 [59.6 ~ 60.3] for those without the therapy). Since the HDL cholesterol level is a component of the formula for calculating ACI, the above dissociating results indicate that lipid‐lowering therapy did not affect ACI directly by altering HDL cholesterol levels. Therefore, lipid‐lowering therapy is unlikely to affect the relationship between ACI and incident hypertension, although the proportion of individuals receiving lipid‐lowering medications increased after the 5‐year follow‐up (Table 1).

This study had several limitations. Underreporting of individual alcohol consumption causes an unavoidable information bias in epidemiological studies [9, 10, 11]. Therefore, it is almost impossible to determine alcohol consumption levels accurately in any cohort. The participants in this study were Japanese men, and further studies are needed to determine whether the ACI is also a useful marker of alcohol intake in women and other ethnic groups. The number of individuals who showed high ACI levels at baseline and low ACI levels five years later was too small to further classify these individuals into more detailed groups. The numbers of the transition groups of regular drinkers at baseline and nondrinkers five years later, heavy drinkers at baseline and nondrinkers five years later, and the 3rd tertile of ACI at baseline and 1st tertile of ACI five years later were smaller than the corresponding other two transition groups for each variable (See the legends to Figures 3, 4, 5); thus, further studies using larger cohorts are needed to confirm the findings of the present study. In this study, age, BMI, medication therapy for hypertension, smoking, and regular exercise were adjusted for in multivariable analyses. However, other possible confounding factors, such as socioeconomic factors, nutrition, and polymorphisms of alcohol‐metabolizing enzymes, may have influenced the relationship between ACI and blood pressure. Information on these factors was not available in this study. Polymorphism of aldehyde dehydrogenase 2 (*ALDH2*) greatly influences drinking behavior and the resulting incidence of AUD in East Asians, including Japanese [24, 25, 26]. Individuals with the inactive form of *ALDH2* (ALDH2*2) show acute symptoms, including palpitations, nausea, and skin flushing, immediately after drinking alcohol, and thus have alcohol intolerance [27, 28]. Accordingly, the numbers of participants in the groups of nondrinkers at baseline and drinkers (regular drinkers and heavy drinkers) after the follow‐up were too small to analyze the relationships of increases in alcohol intake and the tertiles of ACI with changes in blood pressure.

## 5 Conclusions

Although habitual alcohol consumption is confirmed as a risk factor for hypertension, it is difficult to know accurate individual alcohol consumption due to underreporting. ACI tended to be higher with increasing frequency and amount of alcohol consumption and was positively associated with blood pressure and the risk of hypertension. Therefore, ACI is considered to be a useful marker of alcohol consumption for the prevention and treatment of hypertension.

## Author Contributions

I.W. performed all of this study, including Conceptualization, Methodology, Formal analysis, Investigation, Data curation, Original draft preparation and editing of the manuscript, Visualization, and Funding acquisition.

## Funding

This work was supported by Japan Society for the Promotion of Science (25K13516).

## Ethics Statement

This study was approved by the Hyogo College of Medicine Ethics Committee (No. 3 003 in 2020).

## Consent

The database used in this study was supplied by a local health checkup system without individual identification, and no informed consent was obtained from each participant.

## Conflicts of Interest

The author declares no conflicts of interest.

## Data Availability

The data that support the findings of this study are available on request from the corresponding author. The data are not publicly available due to privacy or ethical restrictions.
